# Digital Inclusion, Cultural Capital, and Health Status of Urban and Rural Residents: An Empirical Study Based on 2017 CGSS Database

**DOI:** 10.3390/ijerph20054022

**Published:** 2023-02-23

**Authors:** Zhenyu Sun, Wei Sun, Hongliang Gao, Ruobing Fa, Shaofan Chen, Dongfu Qian

**Affiliations:** 1 School of Health Policy & Management, Nanjing Medical University, Nanjing 211166, China; 2 Chia Tai Tianqing Pharmaceutical Group Co., Ltd., Lianyungang 222000, China; 3 School of Clinical Medicine, Wannan Medical College, Wuhu 241002, China; 4 Center for Global Health, Nanjing Medical University, Nanjing 211166, China

**Keywords:** digital inclusion, cultural capital, health status, influencing mechanism, digital health equity

## Abstract

China is committed to using digital technology to drive urban–rural integration in health care. This study aims to explore the effect of digital inclusion on health status with the mediating role of cultural capital and the digital health disparities between urban and rural residents in China. Using data from the 2017 Chinese General Social Survey (CGSS), the present study adopted an ordinary least squares (OLS) robust standard error regression model to investigate the impact of digital inclusion on health status. In addition, causal step regression (CSR) and bootstrapping methods were combined to test the mediating effect of cultural capital. The results showed that, first, digital inclusion was related to positive and significant effects on resident health status. Second, cultural capital played a mediating role in the relationship between digital inclusion and health status. Third, urban residents gained more health benefits from digital inclusion than rural residents. Additionally, common method variance (CMV) tests, endogenous tests, and a propensity score matching (PSM) analysis showed that the above conclusions remained robust. The government should therefore focus not only on promoting the population’s health by utilizing digital inclusion but also on accelerating digital health equity between urban and rural areas by developing such strategies as a digital infrastructure expansion schedule and digital literacy education and training programs.

## 1. Introduction

Advances in digital technology are accelerating social transformation [1]. The Chinese government has proposed the concept of integrating urban and rural development through digitization, and this involves various sectors of society, such as health, education, and finance [2]. However, few studies have investigated the mechanism of how digitization affects health. In addition, whether digitization promotes or hinders urban–rural integration in health care is still not well understood. Digital inclusion is a concept that consists of digital access, skills, and outputs when considering the digital divide regarding inequality in access to information and communications technology (ICT) and in the ability and benefits of using ICT among those who already have access [3]. Based on the partial understanding of digital inclusion, Kan et al. [4] examined the digital access associations using children’s health status among Chicago families, but no significant correlation was found. The positive and significant effect of Internet use on elderly health with the mediating role of cultural engagement in China was reported by Chen et al. [5]. Kirsten et al. [6] pointed out efficient doctor-patient interactions as a simple output of digital health cutting-edge apps.

Cultural capital is defined as the knowledge and skills that people possess for achieving social advancement and higher status, including being institutionalized, embodied, and objectified [7]. Institutionalized cultural capital is associated with institution-issued educational qualifications and academic credentials, such as diplomas and degrees [7]. Embodied cultural capital is regarded as part of the habitual nature of human beings, such as reading and exercising [8]. Objectified cultural capital refers to the physical possession of transferable cultural products such as books, antiques, and paintings [9]. However, empirical research on cultural capital has never been a fully manipulated concept utilizing indicators of all three types differentiated by Bourdieu [10]. As one non-material form of resource, cultural capital might well be able to determine people’s health status, but studies regarding this issue are few [11]. Gagné et al. [12] showed that cultural capital indicators are fine predictors of adolescent smoking behavior. In addition, the significant and positive effects of cultural capital on physical health and psychological health were revealed by Xu [13] and Kate [14], respectively.

The above literature review shows that most of the existing studies related to the action of digital inclusion and cultural capital on health status have the following three shortcomings. First, limited measuring techniques tend to analyze the association between one dimension of digital inclusion and one aspect of health status. Second, to date, no comprehensive mechanism analyses of the function of digital inclusion, cultural capital, and health status have been carried out. Third, the results and conclusions are not sufficiently robust due to the neglect of variable endogeneity, confounding factors, and methodological biases regarding data collection.

The application of digital technologies reduces the barriers and costs for residents in the process of medical service utilization, such as providing convenient transportation, medical consultation, and payment [15]. It also effectively alleviates the psychological gap caused by the transition of life state [16]. Accordingly, in this study, we make the following assumption:

**H1.**  *Digital inclusion can improve resident health status.*

Residents with strong cultural capital are more capable of using digital devices. This means they can achieve convenient functions, such as health information searching, medical treatment, and interpersonal interactions [3,5], that are considered to be beneficial for their physical health and social adaptability [17]. Based on the cultural capital theory and existing research [9,18], in this study, we make the following assumption:

**H2.**  *Cultural capital mediates the effect of digital inclusion, thereby improving resident health status.*

The gap in Internet facilities, medical care, and education between urban and rural areas in China exists objectively [2,5], and this suggests that rural residents may have a harder time reaping development dividends from the digitization of health. Accordingly, in this study, we make the following assumption:

**H3.**  *Urban residents gain more health benefits from digitization than rural residents.*

The present study aims to utilize data from the 2017 Chinese General Social Survey (CGSS) database to explore the mechanism of how digital inclusion acts on multi-dimensional health, with the mediating role of cultural capital. We further combine this with a heterogeneity analysis of digital health disparities between urban and rural residents in China to investigate whether digitization expedites or impedes urban–rural integration in health care. In addition, we adopt comprehensive post hoc analysis methods to ensure credible results and conclusions. The analyses not only contribute to the literature regarding mechanism studies of the relationship between digitization and health but also provide practical policy suggestions for stimulating Chinese urban–rural integration development in health care.

## 2. Materials and Methods

### 2.1. Data Processing

This study utilized 2017 CGSS data that were collected by the Renmin University of China. The CGSS is the first nationwide, comprehensive, and continuous large-scale social survey project with high-quality data in China. It adopted a multistage stratified sampling method to obtain representative samples and covered all of the provinces except for Hong Kong, Macao, and Taiwan. The 2017 CGSS survey data were released in October 2020 and include 783 variables and 12,582 valid samples. This endeavor is one of the latest projects for studying China’s social issues. We limited the sample group to those who are registered as urban or rural residents (excluding residents without census registration or military service). In addition, after eliminating respondents with missing values and outliers, we ended up with a dataset of 94,626 data points covering 2253 valid samples. The data were obtained using a signed data use agreement from a publicly accessible database at the Renmin University of China Open Research Data platform (http://cgss.ruc.edu.cn/, (accessed on 28 October 2022)) [5].

### 2.2. Variables and Measures

#### 2.2.1. Dependent Variable

According to Huber [19] and Druten et al. [15], we constructed three dependent variables to comprehensively describe the health status of residents, namely, physical health, psychological health, and social adaptability. To define physical health, we utilized the question, “How do you feel about your current state of physical health?”, which had five levels of possible responses ranging from 1 (very unhealthy) to 5 (very healthy). Psychological health was defined using the question, “In the last four weeks, how often have you felt unhappy and depressed?”, and, “In the last four weeks, how often have you felt the difficulties piling up so much that you cannot overcome them?”. Both questions had five possible responses that we redefined as “Never = 5”, “Rarely = 4”, “Sometimes = 3”, “Frequently = 2”, and “Very frequently = 1”. Therefore, the psychological health variable was scored on a scale of 2–10, with higher scores associated with a better psychological health status. Social adaptability was measured using the questions, “In the last 12 months, how often have you participated in activities organized by a leisure group, sports group, or cultural group?”, “In the last 12 months, how often have you participated in activities organized by a political party, political group, or political society?”, and “In the last 12 months, how often have you volunteered for a charity or religious organization?”. The above three questions had five possible responses: “Once a week or more”, “One to three times a month”, “Several times last year”, “One-time last year”, and “Never”. We redefined “Never = 0” and “Otherwise = 1”. Accordingly, the social adaptability variable was scored on a scale of 0–3, with higher scores indicating a stronger social adaptation ability.

#### 2.2.2. Independent Variable

The independent variable in this study was digital inclusion, and this was divided into digital access, digital skills, and digital output according to Riggins and Dewan [3]. Digital access was measured using the question, “Do you have Internet access at home?”. The available answers were edited to “No = 0” or “Yes = 1”. The measurement of digital skills included the respondents’ self-report on six common operating abilities of digital devices including “using a computer to open websites”, “using a smartphone to install apps”, and “using the Internet to find the information they want”. Each of the six questions originally had five answers that we recorded as “Poor = 0”, “General = 1”, and “Good = 2”. Digital outputs were assessed based on the responses of the respondent’s use of digital tools in six related activities to questions such as “In the past year, how often have you used the Internet (e.g., WeChat, Microblog, and Twitter) for self-presentation?” and “In the past year, how often have you used the Internet for business transactions (e.g., transferring accounts, making payments, and shopping)?”. The six questions in the original questionnaire primarily had five optional answers: “Never”, “Rarely”, “Sometimes”, “Often”, and “Always”, and these were enumerated as “Poor = 0”, “General = 1”, and “Good = 2”. Finally, the scores of the three dimensions, i.e., digital access, skills, and output, were summed. The score range of digital inclusion was from 0 to 25. The higher the score, the higher the degree of digital inclusion.

#### 2.2.3. Mediating Variable

The mediating variable in this study was cultural capital that consists of institutionalized and embodied cultural capital by reference to the research of Tilbrook et al. [20] Educational level was used as the proxy variable of institutionalized cultural capital [7]. The question was “What is your highest level of education?” in the original questionnaire, including 13 options for the educational level, and this was reprocessed and merged into “Not attended school = 0”, “Primary school or below = 1”, “Junior high school = 2”, “Senior high or Technical secondary school = 3”, “Junior or Undergraduate school = 4”, and “Graduate school or above = 5”. Embodied cultural capital is typically reflected by the frequency of individual cultural engagements [8]. The current study was based on the question, “In the past year, did you often engage in the following activities in your free time?”, and the options included 10 cultural activities such as “Going out to the movies”, “Taking part in physical exercise”, and “reading books/newspapers/magazines” as observational indicators. We also included five additional options: “Every day”, “Several times a week”, “Several times a month”, “Several times a year or less”, and “Never” in the original questionnaire. These were re-edited as “Never = 0” and “Otherwise = 1”. Thus, cultural capital was measured by the sum of the scores of the above two dimensions that ranged from 0 to 15. The higher the score, the stronger the cultural capital.

#### 2.2.4. Control Variable

The previous research conclusions indicated that sociodemographic characteristics would significantly affect the multi-dimensional health of residents [18]. We then selected nine sociodemographic variables (living region, census register, gender, age, race, marital status, religion, family income, and working status) as the control variables. It should be noted that we utilized the respondents’ subjective feelings on household economic status as the measurement of the family income variable based on the principle of utility economics [21]. This subjective feeling is an individual’s evaluation of whether he or she is poor or rich, and this self-evaluation standard is typically associated with the reference group set by the individual himself or herself that avoids the drawback of using a certain percentage of the median household income as the threshold standard to distinguish the family income level [22]. Table 1 presents a descriptive statistical analysis of all of the variables.

### 2.3. Statistical Analysis

#### 2.3.1. The Mediation Effect Test Model

**Figure 1:**
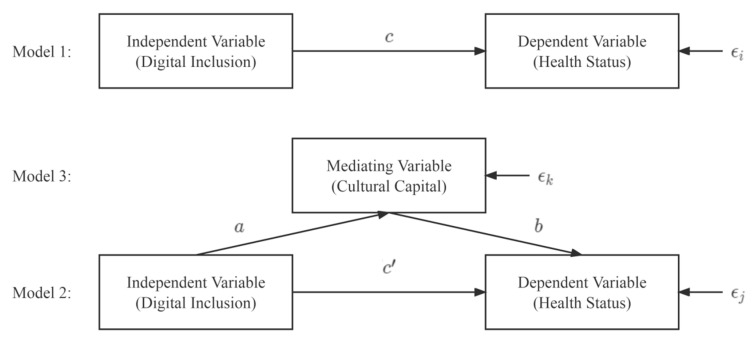
The basic principles of mediation effect testing.

Based on the relevant research of Zhang et al. [23] combined with the research objects of this study, the mediation effect test model was constructed as follows:

In the following three models, ${Health}_{i,j,k}$ represents the dependent variable “health status”, ${Digital}_{i,j,k}$ represents the independent variable “digital inclusion”, ${Cultural}_{j,k}$ represents the mediating variable “cultural capital”, ${Control}_{i,j,k}$ represents the control variables, $\varepsilon_{i,j,k}$ represents the random error term of the regression models, and $a,b,c,c^{\prime },\beta ,\beta^{\prime },\beta^{\prime \prime }$ are the coefficient vectors. The basic principles of the mediation effect testing are shown in Figure 1.

Model 1: ${Health}_{i}=c{Digital}_{i}+\beta {Control}_{i}+\varepsilon_{i}$

Model 1 is a regression analysis of the independent variable “digital inclusion” and the dependent variable “health status” with the ordinary least squares (OLS) robust standard error regression model. This aims to obtain the total effect value *c*.

Model 2: ${Health}_{j}=c^{\prime }{Digital}_{j}+b{Cultural}_{j}+\beta^{\prime }{Control}_{j}+\varepsilon_{j}$

Model 2 is a regression analysis of the independent variable “digital inclusion”, the mediating variable “cultural capital”, and the dependent variable “health state” with the aim of obtaining the direct effect value *c*′ and the intermediate effect process value *b*. The difference between Model 2 and Model 1 is that Model 2 adds the mediating variable “cultural capital” based on Model 1.

Model 3: ${Cultural}_{k}=a{Digital}_{k}+\beta^{\prime \prime }{Control}_{k}+\varepsilon_{k}$

Model 3 is a regression analysis of the independent variable “digital inclusion” and the mediating variable “cultural capital” to acquire the intermediate effect process value *a*.

After referring to the research of Chen et al. [5,24,25], we chose to use both the causal step regression (CSR) and bootstrap methods to execute the mediating effect test procedure.

#### 2.3.2. Post Hoc Analyses

Variable endogeneity, confounding factors, and methodological biases regarding data collection will lead to less robust research results and conclusions. We therefore utilized the appropriate post hoc analyses to avoid this. First, since all of the variables were measured by surveying Chinese mainland residents, our findings might be biased by the common method variance (CMV). As suggested by Singh-Manoux [26] and Sören Fiedler [27], we conducted a Harman’s single-factor test and confirmatory factor analysis (CFA) to assess the CMV.

Second, when used as explanatory variables, endogenous variables will lead to biased regression estimates because of the correlation with missing or unknown omitted variables [28]. Following common practice [29,30], we selected the Internet access of the respondents lagged by one period (i.e., whether the respondents used the Internet frequently in the last year) as an instrumental variable (IV), and sociodemographic characteristics, such as age, gender, and race, were utilized as control variables. In addition, the endogeneity of digital inclusion in the model for each health status outcome variable was tested using the Durbin–Wu–Hausman (DWH) test.

Third, some confounding factors may affect the population’s digital inclusion and health status simultaneously. In addition, the data bias could lead to the problem of inconsistent estimates of the real situation. We performed the method of propensity score matching (PSM) to solve this. Accordingly, we divided residents into “high digital inclusion” (scores ≥ 13) and “low digital inclusion” (scores < 13) groups based on their scores on the digital inclusion variable. We also utilized a nearest-neighbor matching model to calculate the average treatment effect on the treated (ATT) participants by referring to the studies of Sun [31] and Hou et al. [21].

All of the statistical analyses were performed using SPSS (version 25.0) [32] and Stata SE (version 15.0) [33]. A *p* of 0.05 was considered to be significant.

## 3. Results

### 3.1. Characteristics of the Samples

More than half of the population (1255, 55.70%) were from eastern China. The number of rural residents (1173, 52.06%) was similar to that of urban residents (1080, 47.94%). The proportion of female (1127, 50.02%) and male (1126, 49.98%) groups was relatively close. There were 1348 (59.83%) respondents aged 44 or younger, and 143 (6.35%) respondents were aged 65 or older. The Han population (2107, 93.52%) was dominant. A total of 1692 (75.1%) respondents were “married with spouse”, and 561 (24.90%) respondents were not “married with spouse” (i.e., widowed or divorced). Of all of the respondents, 2057 (91.30%) were nonreligious, and 196 (8.70%) were religious. For the family income group, 1234 (54.77%) respondents considered they were average, 820 (36.40%) thought they were poor, while only 199 (8.83%) said they were wealthy. A total of 747 (33.16%) respondents were jobless, and 1506 (66.84%) were employed. Table 2 shows the demographic characteristics of the respondents.

### 3.2. OLS Analysis (Model 1)

We first tested the multicollinearity to avoid its negative impact on the results of the OLS model. The results showed that the variance inflation factor (VIF) values of each variable were all less than two (the cut-off value was five), indicating there was no multicollinearity problem in this study. Second, we conducted a White test, and the results showed that the OLS model had a heteroscedasticity problem. Thus, the OLS robust standard error regression model was adopted for our OLS analysis. The OLS analysis results showed that the regression coefficients of digital inclusion on physical health, psychological health, and social adaptability were 0.019 (*p* < 0.001), 0.017 (*p* < 0.001), and 0.033 (*p* < 0.001), respectively, reflecting a significant positive relationship between digital inclusion and health status, and H1 was verified. Table 3 shows the correlation between the digital inclusion and resident health status while controlling for the other relevant variables.

### 3.3. CSR Analysis (Models 2 and 3)

The results of the CSR showed that the coefficient of digital inclusion on cultural capital was 0.184 (*p* < 0.001). The regression coefficients of cultural capital on physical health and social adaptability were 0.019 (*p* < 0.05) and 0.124 (*p* < 0.001), respectively, while cultural capital had no significant effect on physical health. In addition, compared with the OLS regression results, the coefficient values of digital inclusion on physical health (0.019 vs. 0.016) and social adaptability (0.033 vs. 0.010) both showed a decrease. Therefore, we could preliminarily determine that cultural capital possessed a significant mediating effect on the association between digital inclusion and health status. Table 4 shows the test results of the mediating effect of cultural capital based on the CSR model.

### 3.4. Bootstrap Analysis (Model 2)

**Figure 2:**
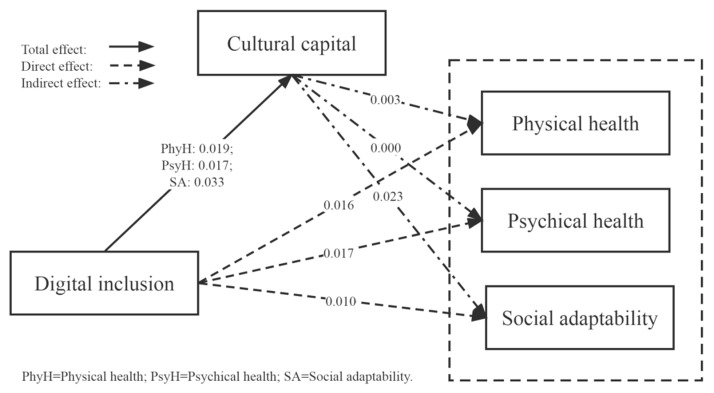
The impact of digital inclusion on health status: the mediating effect of cultural capital.

Based on the above results, we further tested the mediation effect using the bootstrap method. The results showed that the direct and indirect effect coefficients of digital inclusion on physical health were 0.016 [95% CI, 0.008–0.024] and 0.003 [95% CI, 0.003–0.042], respectively. The mediating effect value of cultural capital in the influence of digital inclusion on psychological health was zero. The direct effect coefficient of digital inclusion on social adaptability was 0.010 [95% CI, 0.002–0.018], and the indirect effect coefficient was 0.023 [95% CI, 0.128–0.177]. Thus, in general, cultural capital played a mediating role in the relationship between digital inclusion and resident health status, and H2 was verified. Table 5 shows the results of testing the cultural capital intermediary mechanism using the bootstrap method. Figure 2 shows the diagram of the mediating effect of cultural capital based on the bootstrap analysis results.

### 3.5. Urban–Rural Heterogeneous Analysis

As shown in Table 6, rural residents were slightly better than urban residents in physical health (mean values 3.82 vs. 3.78); however, their psychological health (7.97 vs. 8.22), social adaptability (0.60 vs. 0.91), digital inclusion (15.75 vs. 17.60), and cultural capital (8.35 vs. 10.39) were all inferior to urban residents. Moreover, the positive effects of digital inclusion on the physical health (coefficient value 0.023, *p* < 0.001 vs. 0.015, *p* < 0.05) and psychological health (0.018, *p* < 0.05 vs. 0.013, *p* < 0.1) of urban residents were significantly higher than that on the physical health and psychological health of rural residents after controlling for other relevant variables, and H3 was verified. Table 7 shows the gain divergences in digital inclusion for promoting health between urban and rural residents.

### 3.6. Post Hoc Analyses

#### 3.6.1. Common Method Variance

The results of the Harman’s single-factor test showed that there was no general factor accounting for more than 20% of the variation. The CFA results showed that the values of *X*^2^/*df* = 38.05, GFI = 0.88, RMSEA = 0.13, RMR = 1.24, CFI = 0.51, NFI = 0.51, and NNFI = 0.39 were not up to the standard. The above evidence collectively suggested that the CMV was not a valid threat in this study.

#### 3.6.2. Endogeneity Issues

The DWH test results provided no evidence of endogeneity of digital inclusion in the OLS models; that is, the results of the prior OLS robust standard error regression models were acceptable.

#### 3.6.3. Data Bias and Confounding Factor Interference

The results of the PSM analysis showed that the degree of digital inclusion significantly increased the probability of physical health (ATT = 0.291, *p* < 0.001), psychological health (ATT = 0.413, *p* < 0.001), and social adaptability (ATT = 0.518, *p* < 0.001). Evidence from the above PSM analysis indicated that the conclusion that digital inclusion improves health status based on the OLS regressions was robust.

## 4. Discussion

This study utilized the 2017 CGSS data to probe the effect of digital inclusion on multi-dimensional health, with the mediating role of cultural capital and the digital health disparities between urban and rural residents in China. We utilized the OLS robust standard error regression model to understand how digital inclusion would affect the three types of health of Chinese residents. To explore the mechanism of the impact of digital inclusion on health, we utilized cultural capital as a mediating variable for mediating the effect analysis. Moreover, we performed a heterogeneity analysis of digital health disparities between urban and rural residents. The CFA, IV, and PSM methods were adopted for the CMV, endogenous, and robustness tests, respectively, to verify the analysis. The systematic and rigorous findings are presented as follows:

### 4.1. Digital Inclusion Is a Health Booster

The results of the OLS analysis showed that, first, digital inclusion had a positive impact on physical health, and this was consistent with a previous study [34]. Second, digital inclusion significantly and positively affected psychological health, which was also similar to previous findings [35,36]. Third, digital inclusion was significantly, positively, and strongly associated with social adaptability, and this was similar to the results found in the prior work of Jones [37] and Ghouse et al. [38].

In summary, the higher the degree of digitization that people were engaged with, the greater the influence on physical and psychological health and the greater their social adaptability would be. Thus, overall, digital inclusion can effectively improve the health status of the population, and our post hoc analyses confirmed the robustness of this conclusion. A possible reason for this result may be that the information and resources on the Internet are more abundant, and this further empowers people to adopt healthy behaviors and keep connected to their social networks [5]. Furthermore, Keesara et al. [39] argued that digital form services maintained health care operations and safeguarded population health while reducing face-to-face encounters, and Hila et al. [40] showed how digital networks increase the subjective well-being of participants in the COVID-19 pandemic context.

### 4.2. Cultural Capital Mediated the Effect of Digital Inclusion for Promoting Health

Our findings demonstrated that cultural capital was introduced as a mediating variable to identify the influencing mechanism of digital inclusion on the three dimensions of health. We found that digital inclusion affected cultural capital positively and significantly. This may because digital inclusion will increase educational opportunities and cultural engagement activities [17]. Digitization can dramatically reduce the costs of search, replication, transportation, tracking, and the verification of information [41]. In addition, people are able to use digital equipment as a medium to obtain information regarding cultural activities at nearly zero cost, and this will further promote people’s accumulation of cultural capital [42].

However, we did notice significant positive correlations between cultural capital and the level of physical health and social adaptability, and these were also demonstrated in previous studies [43]. People who are highly educated and frequently involved in cultural activities may have greater physical exercise consciousnesses and richer social networks [44] and therefore possess a superior physical health status and social adaptability. Additionally, Leonhardt et al. [45] pointed out people with superior cultural capital are more likely to withstand the shock of the COVID-19 pandemic. Khawaja [46] and Saville et al. [47] reported the positive effects of cultural capital on psychological health, while the research results of Pan [11] and Pinxten et al. [48] suggested that cultural capital was not related to psychological health. There was no evidence found in this study that cultural capital was beneficial or unfavorable for psychological health.

### 4.3. Digital Inclusion Risks Exacerbating Health Inequities between Urban and Rural Populations

Based on the results of the urban–rural heterogeneity analysis, we found that the health disparities between urban and rural residents not only lay in the fact that the capital endowments [49] (e.g., family income, cultural capital, and digital inclusion level) of rural residents were poorer than those of urban residents but also in the fact that urban residents could gain more health benefits from digitization than rural residents. These findings are a reminder of the fact that digital inclusion exacerbates the risk of health inequities between urban and rural areas and may run counter to the Chinese government’s desire to promote the development of urban–rural integration in health care via digitization, that is, the digital health inequity between urban and rural areas.

Furthermore, Haenssgen [50] pointed out that rapid mobile phone diffusion created opportunities to improve people’s access to health care in rural India, but it also created new forms of marginalization among poor rural households. Chen et al. [5] indicated that the effect of Internet use on the physical and mental health of Chinese elderly in agricultural households was significantly lower than that on their counterparts in the non-agricultural group. Kaihlanen et al. [51] found that the COVID-19 pandemic has given an unprecedented boost to already increased digital health services, while hampering vulnerable groups’ access to digital health services due to insufficient digital facilities and skills. The studies conducted by the aforementioned scholars indirectly support one of our views that digitization risks tearing apart urban and rural integrated development in health care if the authorities do not utilize pro-rural policy interventions related to digital health in a timely manner, especially in the context of COVID-19 pandemic.

Given the above empirical analysis results, we propose the following pro-rural policy recommendations: first, build a favorable rural digital environment by constructing digital infrastructures (e.g., broadband router and Wi-Fi Internet), providing subsidies to rural residents for purchasing smartphones, and offering preferential Internet charges [52]; second, develop digital education and training programs to enhance digital skills, awareness, and the literacy of rural residents [53]; third, provide financial support for encouraging local communities to organize various forms of cultural activities among rural residents to motivate them to participate in such cultural activities [5].

### 4.4. Strengths and Limitations

This study enriches and extends the previous research and contributes to the literature in the following aspects. First, we divided health status into three dimensions (physical health, psychological health, and social adaptability) in our practical evaluation, providing a multi-dimensional application of health connotation. Second, our findings provide empirical evidence for the first time that cultural capital plays an important mediating role between digital inclusion and the health of Chinese residents. Third, from an innovative research perspective, our results revealed the causes of digital health inequity between urban and rural residents in terms of capital endowment and digital benefit disparities simultaneously. Additionally, we strengthened the reliability of the results using systematic post hoc analyses.

Although potentially useful for guiding the government’s use of digitization to drive urban–rural integration in health care, our study has several data-related limitations. First, our data are five years old now, even though we utilized the latest data from the CGSS database. Second, we only utilized the data from the one period and did not consider variable-related changes with time. Third, there exist shortcomings in the measurements of digital inclusion and cultural capital due to limitations in the availability of the secondary data variables. Fourth, important control variables, such as medical insurance participation, were ignored because of data restrictions. However, these limitations will provide research directions for further studies in the future.

## 5. Conclusions

This study unveiled that digital inclusion indeed contributes directly to improving the health of both urban and rural people in China and explained the mechanism of how digital inclusion affects multi-dimensional health status under the mediating role of cultural capital. In addition, our empirical results indicated that the reason for the digital health inequity between Chinese urban and rural residents was not only because urban residents possess more powerful capital endowment but also because urban residents obtain more health gains from digitization than rural residents. The above findings provide a reliable and practical basis for authorities to formulate pro-rural policies on digital health care facility construction and literacy education as well as to formulate other plans to narrow the health gap between urban and rural areas by using digitization.

## Figures and Tables

**Table 1:** Definition and descriptive statistics of various variables.

Variables	Category	Descriptions	Mean	Standard Deviation
Dependent variables	Physical health	Ranging from 1 to 5	3.80	0.973
	Psychical health	Ranging from 2 to 10	8.09	1.365
	Social adaptability	Ranging from 0 to 3	0.75	0.934
Independent variables	Digital inclusion	Ranging from 0 to 25	16.64	6.201
Mediating variables	Cultural capital	Ranging from 0 to 15	9.33	2.821
Control variables	Living region	Eastern area = 1, Central area = 2, Western area = 3	1.64	0.796
	Census register	0 = Rural, Urban = 1	0.48	0.500
	Gender	Female = 0, Male = 1	0.50	0.500
	Age	Ranging from 18 to 86	41.40	14.012
	Race	Minority = 0, Han = 1	0.94	0.246
	Marital status	Not “married with spouse” = 0, Married with spouse = 1	0.75	0.433
	Religion	Nonreligious = 0, Religious = 1	0.09	0.282
	Family income	Poor = 1, General = 2, Rich = 3	1.72	0.614
	Working status	Jobless = 0, Working = 1	0.67	0.471

**Table 2:** The characteristics of the sample (N = 2253).

Variables	Category	N	Percentage (%)
Living region	Eastern area	1255	55.70
	Central area	543	24.10
	Western area	455	20.20
Census register	Rural	1173	52.06
	Urban	1080	47.94
Gender	Female	1127	50.02
	Male	1126	49.98
Age	From 18 to 44	1348	59.83
	From 45 to 64	762	33.82
	At least 65	143	6.35
Race	Minority	146	6.48
	Han	2107	93.52
Marital status	Not “married with spouse”	561	24.90
	Married with spouse	1692	75.10
Religion	Nonreligious	2057	91.30
	Religious	196	8.70
Family income	Poor	820	36.40
	Average	1234	54.77
	Good	199	8.83
Working status	Jobless	747	33.16
	Working	1506	66.84

**Table 3:** The OLS analysis results.

Variables	Physical Health		Psychical Health		Social Adaptability	
	*β*	*p*-Value	*β*	*p*-Value	*β*	*p*-Value
Digital inclusion	0.019 ***	<0.001	0.017 ***	0.004	0.033 ***	<0.001
Living region	−0.086 ***	0.001	−0.251 ***	<0.001	0.104 ***	<0.001
Census register	−0.017	0.67	0.020	0.74	0.213 ***	<0.001
Gender	0.115 ***	0.003	0.113 **	0.05	0.108 ***	0.005
Age	−0.019 ***	<0.001	0.012 ***	<0.001	0.006 ***	0.003
Race	0.047	0.56	−0.166	0.17	−0.079	0.29
Marital status	0.084 *	0.07	0.300 ***	<0.001	−0.146 ***	0.004
Religion	0.029	0.69	−0.102	0.35	0.169 **	0.01
Family income	0.284 ***	<0.001	0.220 ***	<0.001	0.242 ***	<0.001
Working status	0.168 ***	<0.001	−0.144 **	0.02	−0.013	0.76
Constant terms	3.628 ***	<0.001	7.333 ***	<0.001	−0.601 ***	<0.001
Sample size	2253		2253		2253	
R^2^	0.179		0.077		0.108	

Abbreviations: *β*, standardized beta regression coefficient. * *p* < 0.1, ** *p* < 0.05, *** *p* < 0.01.

**Table 4:** The test of the mediating effect of cultural capital based on the CSR model.

Variables	Cultural Capital		Physical Health		Psychical Health		Social Adaptability	
	*β*	*p*-Value	*β*	*p*-Value	*β*	*p*-Value	*β*	*p*-Value
Cultural capital	NA		0.019 **	0.03	0.001	0.91	0.124 ***	<0.001
Digital inclusion	0.184 ***	<0.001	0.016 ***	<0.001	0.017 ***	0.007	0.010 **	0.01
Control variables	Yes		Yes		Yes		Yes	
Constant terms	5.069 ***	<0.001	3.533 ***	<0.001	7.326 ***	<0.001	−1.229 ***	<0.001
Sample size	2253		2253		2253		2253	
R^2^	0.394		0.181		0.077		0.193	

Abbreviations: NA, not applicable; *β*, standardized beta regression coefficient. ** *p* < 0.05, *** *p* < 0.01.

**Table 5:** The test of the mediating effect of cultural capital based on the Bootstrap method.

Variables	DI→(CC→) PhyH		DI→(CC→) PsyH		DI→(CC→) SA	
	Coefficient	95% CI	Coefficient	95% CI	Coefficient	95% CI
Direct effect	0.016	[0.008–0.024]	0.017	[0.005–0.029]	0.010	[0.002–0.018]
Indirect effect	0.003	[0.003–0.042]	0.000	[−0.018–0.024]	0.023	[0.128–0.177]
Total effect	0.019	[0.012–0.027]	0.017	[0.006–0.028]	0.033	[0.025–0.040]
Proportion of mediating effect	17.88%		0		69.81%	

Abbreviations: CI = confidence intervals; DI = Digital inclusion; CC = Cultural capital; PhyH = Physical health; PsyH = Psychical health; SA = Social adaptability.

**Table 6:** The health status, digital inclusion, and cultural capital gap between urban and rural residents.

Variables	Rural (N = 1173)				Urban (N = 1080)			
	Mean	Standard Deviation	Minimum	Maximum	Mean	Standard Deviation	Minimum	Maximum
Physical health	3.82	1.006	1	5	3.78	0.937	1	5
Psychical health	7.97	1.314	5	10	8.22	1.379	4	10
Social adaptability	0.60	0.871	0	3	0.91	0.974	0	3
Digital inclusion	15.75	6.523	0	25	17.60	5.681	0	25
Cultural capital	8.35	2.808	1	14	10.39	2.423	2	15

**Table 7:** The disparities of digital inclusion’s effects on health status between urban and rural residents.

Variables	Physical Health				Psychical Health				Social Adaptability			
	Urban		Rural		Urban		Rural		Urban		Rural	
	*β*	*p*-Value	*β*	*p*-Value	*β*	*p*-Value	*β*	*p*-Value	*β*	*p*-Value	*β*	*p*-Value
Digital inclusion	0.023 ***	<0.001	0.015 **	0.01	0.018 **	0.05	0.013 *	0.08	0.034 ***	<0.001	0.034 ***	<0.001
Control variables	Yes		Yes		Yes		Yes		Yes		Yes	
Constant terms	3.357 ***	<0.001	3.913 ***	<0.001	7.401 ***	<0.001	7.359 ***	<0.001	−0.373	0.15	−0.635 ***	0.002
Sample size	1080		1173		1080		1173		1080		1173	
R^2^	0.191		0.179		0.073		0.067		0.085		0.091	

Abbreviations: *β*, standardized beta regression coefficient. * *p* < 0.1, ** *p* < 0.05, *** *p* < 0.01.

## Data Availability

The datasets generated and/or analyzed in the current study are available in the Chinese General Social Survey Database (2017 version, http://cgss.ruc.edu.cn/, (accessed on 28 October 2022)). All the data used in this study are available to the public, and hence, no ethical or governmental permissions were required for this study.
